# Computational Approaches for Drug Discovery

**DOI:** 10.3390/molecules24173061

**Published:** 2019-08-22

**Authors:** Simone Brogi

**Affiliations:** Department of Pharmacy, University of Pisa, via Bonanno 6, 56126 Pisa, Italy; simone.brogi@unipi.it; Tel.: +39-05-0221-9613

Computational approaches represent valuable and essential tools in each step of the drug discovery and development trajectory. Several computational methodologies are suitable to help researchers in the identification and investigation of new drug candidates. These in-silico procedures include: Virtual Screening [1,2,3,4], 3D-QSAR (Three-Dimensional Quantitative Structure-Activity Relationship) [5,6,7,8], Molecular Docking studies [9,10,11,12,13,14,15], Molecular Dynamics simulations [16,17], and the prediction of ADME+T properties [18,19,20]. Accordingly, we realized a Special Issue enclosing several papers highlighting the most recent advances about the in-silico methods employed in the field of drug discovery and design.

The Special Issue focused on Computational Approaches for Drug Discovery comprises 13 Original Research Articles, 1 Communication, and 1 Review. The Original Research Articles describe the application of several computational methods including quantitative structure-activity/toxicity relationships (QSAR and QSTR, respectively), virtual screening, pharmacophore modelling, ab-initio calculation, molecular docking, and Molecular Dynamics simulation. In addition, a new method for generating 3D pharmacophore models and a novel rescoring function for identifying bioactive peptides have been described in two articles of the mentioned special issue.

In particular, Wang and coworkers reported a combined in-silico method for predicting potential drug targets of aconitine alkaloids that are involved in cardiotoxicity. This method allowed to investigate the QSTR of these compounds, leading to a better insight into the cardiotoxicity induced by the compounds that have similar structures with respect to their derivatives. This procedure appears to be useful for following structural modifications of the aconitine alkaloids for the design of improved derivatives [21]. Zhang and collaborators implemented a 3D-QSAR method in a virtual screening protocol in order to discover a novel ligand potentially able to inhibit HIV-1 entry and infection via CD4. In this paper, biological and computational analyses using bioactivity evaluation, Rule of Five (RO5), comparative molecular field analysis (CoMFA)/comparative molecular similarity index analysis (CoMSIA) models, and 3D-QSAR allowed to identify the derivative **3** as a promising lead compound for the further development of therapeutics targeting HIV-1 entry [22]. Also, Li and colleagues reported a virtual screening protocol that allowed to identify a small set of compounds potentially able to inhibit human topoisomerase I (Top1) protein. They applied random forest (RF), support vector machine, k-nearest neighbor, and C4.5 decision tree for establishing classification models for evaluating if an unknown molecule could be an inhibitor of human Top1protein. Although these models have achieved satisfactory results, through comparative analysis it was found that the RF model showed a better forecasting effect. So, the parameters were further optimized to generate the best-performing RF model. The resulting model was employed in a ligand-based virtual screening using Maybridge database. After that, the retrieved molecules were docked against Top1. Finally, six top-ranked molecules were screened out and a common backbone which is entirely different from that of existing Top1 inhibitors reported in literatures was found [23]. Flores-Sumoza and co-workers carried out a classical QSAR analysis, combined with docking simulation, of a series of 4-pyridone derivatives as antimalarial agents. The minimum energy structures of 22 derivatives have been optimized at Density Functional Theory level, and several quantum molecular descriptors, including electronic and thermodynamic descriptors, were computed for the mentioned derivatives in order to obtain a statistical and meaningful QSAR equation. Following this computational protocol, promising compounds acting as antimalarial agents were selected [24]. These studies demonstrated that coupling structure- and ligand-based techniques is particularly useful for identifying novel compounds with interesting biological activities against a given target.

The combination of different in-silico techniques is the focus of the paper authored by Bittencourt and colleagues. In fact, in this article, the integration of diverse in-silico techniques (molecular docking; molecular dynamics; thermodynamic profiles; and the prediction of oral bioavailability, bioactivity and toxicity) was employed for searching new anti-inflammatory drugs, acting as COX-2 inhibitors, with better pharmacokinetic and toxicological profiles with respect to the available drugs. This procedure was useful for selecting compounds with satisfactory drug-like and anti-inflammatory profile with respect to the commercial compound [25]. Borges and colleagues investigated the anti-inflammatory profile of a series of phenylbutazone derivatives with the aim to identify compounds with a better pharmacological profile with respect to the phenylbutazone. In particular, by combining quantum chemistry calculations, docking studies and toxicological predictions, few phenylbutazone derivatives have been selected for their potential in inhibiting human as well as murine COX-2 and for their safer profile with respect to the phenylbutazone. The results can explain the biological properties of phenylbutazone and support the design of potentially safer candidates [26]. The anti-inflammatory activity of hypericin, the most abundant metabolite of *Hypericum perforatum L.* (St. John’s Wort), was investigated by Dellafiora and co-workers employing several molecular modelling approaches including docking simulations, pharmacophoric modeling, and molecular dynamics. By combining these computational techniques, it has been highlighted that hypericin can behave as an inhibitor of janus kinase 1, a relevant enzyme in inflammatory response. In particular, the in-silico study estimated the capability of molecules (hypericin and some of its analogues) to interact and persist within the enzyme pocket. The results highlighted the capability of hypericin, and some of its analogues and metabolites, to behave as ATP-competitive inhibitors providing: (i) a likely mechanistic elucidation of anti-inflammatory activity of *H. perforatum* extracts containing hypericin and related compounds; and (ii) a rational-based prioritization of *H. perforatum* components to further characterize their actual effectiveness as anti-inflammatory agents [27]. Another interesting paper authored by de Oliveira Araújo and colleagues reported the computational investigation of a small set of bisphosphate-based derivatives as inhibitors of 3-deoxy-D-manno-octulosonate 8-phosphate (KDO8P) synthase, which is a key enzyme for the lipopolysaccharide (LPS) biosynthesis of Gram-negative bacteria and a potential target for developing new antimicrobial agents. In this work, computational methods were used to generate a homology model of KDO8P synthase from Neisseria meningitides and to investigate the molecular mechanism of its inhibition, by means of molecular docking studies and molecular dynamics investigation, by three bisphosphate inhibitors: BPH1, BPH2, and BPH3 [28]. SAR analysis was performed by Lopez-Lopez and colleagues taking into account the insights from activity landscape, docking and molecular dynamics for understanding the profile of dual inhibitors of major epigenetic targets: lysine metiltransferase (G9a) and DNA metiltranferase 1 (DNMT1). The activity landscape analysis of 50 published compounds with reported experimental activity for both targets, revealed the presence of activity cliffs (pairs of compounds with high structure similarity but large activity difference), leading to the identification of interactions with key residues involved in the dual activity or selectivity with the epigenetic targets [29]. 

In the field of dual active compounds, Kowal and collaborators employed a novel approach for searching molecules able to interact with acetylcholinesterase (AChE) and the α7 nicotinic acetylcholine receptor (nAChR) for developing novel compounds for the potential treatment of neurodegenerative disorders. Using the homology modeling technique for generating a model for α7 nAChR and the crystal structure of AChE, a high throughput virtual screening protocol was adopted for identifying potential dual AChE/α7 nAChR ligands. Starting from 87,250 compounds from a ZINC database of natural products and their derivatives, a subset of potential dual AChE/α7 nAChR ligands was tested in vitro using the colorimetric method of Ellman and two-electrode voltage-clamp electrophysiology, respectively. Following this protocol, two promising compounds with dual activity in the micromolar range were identified This study represents the first report in which the use of computational procedures allowed the identification of compounds with dual inhibitory activity against both the AChE enzyme and the α7 nAChR, confirming that computational methods can be a valuable tool in the early lead discovery process [30]. The ab-initio calculation coupled to molecular docking simulation has been used by Da Silva Costa and co-workers to evaluate the antioxidant potential of two caffeine analogs (ZINC08706191 (Z91) and ZINC08992920 (Z20)). Molecular docking, quantum chemical calculations (HF/6-31G**) and Pearson’s correlation have been performed. Molecular docking results of Z91 and Z20 showed both the lower binding affinity (BA) and inhibition constant (Ki) values for the receptor–ligand interactions in the three tested enzymes (Cytochrome P450—CYP450, Myeloperoxidase—MP and NADPH Oxidase—NO) than the control molecules (5-fluorouracil—FLU, melatonin—MEL and dextromethorphan—DEX, for each receptor respectively). Molecular descriptors were correlated with Ki and strong correlations were observed for the CYP450, MP and NO receptors. These and other results attest the significant antioxidant ability of Z91 and Z20, that may be indicated for further analyses in relation to the control of oxidative stress and as possible antioxidant agents to be used in the pharmaceutical industry [31]. Frau and collaborators employed an in-silico protocol to assess the reactivity behavior of the anticancer marine drugs, Soblidotin and Tasidotin based on the calculation of the global and local descriptors resulting from Chemical Reactivity Theory (CRT), also known as Conceptual DFT, for their consideration as a useful complement to approximations based on Molecular Docking. The information on the global and local reactivity descriptors of the Soblidotin and Tasidotin molecules, obtained through our proposed methodology, may be used for the design of new pharmaceutical analogs by relying on the chemical interactions between these peptides and their receptors. It can be concluded that the CRT approximation to the global and local chemical reactivity, based on the descriptors, can provide interesting information for the consideration of both molecules as potential therapeutic drugs. This is complemented by a study on Advanced Glycation Endproduct (AGE) inhibition, by comparison with the usual molecular systems considered for the task, as a re-purposing study. Finally, the bioactivity scores for Soblidotin and Tasidotin are predicted through an empirical procedure, based on comparison with molecular structures with well-known pharmacological properties [32].

The next two articles illustrated novel methodologies for: i) generating pharmacophore models using 3D pharmacophore signatures and ii) identifying new bioactive molecules from short peptide libraries using PeptoGrid, a rescoring function for AutoDock Vina. In particular, Kutlushina and collaborators developed a new approach to 3D pharmacophore representation and matching which does not require pharmacophore alignment. Since there are just a few free ligand-based pharmacophore modeling tools and these have a lot of restrictions, e.g., using a template molecule for alignment, this tool can be relevant in the pharmacophore modeling field. The approach searches for 3D pharmacophore models starting from 2D structures of available active and inactive compounds. The implemented approach was successfully applied for several retrospective studies. The results were compared to a 2D similarity search, demonstrating some of the advantages of the developed 3D pharmacophore models. Also, the generated 3D pharmacophore models were able to match the 3D poses of known ligands from their protein–ligand complexes, confirming the validity of the models. The developed approach is available as an open-source software tool: http://www.qsar4u.com/pages/pmapper.php and https://github.com/meddwl/psearch [33]. Zalevsky and co-workers reported in a communication the development of a novel algorithm PeptoGrid that rescores poses predicted by AutoDock Vina according to the frequency information of ligand atoms with particular properties appearing at different positions in the target protein’s ligand-binding site. This tool can be useful in the field of bioactive peptides. In fact, peptides are promising drug candidates due to high specificity and standout safety. The identification of bioactive peptides using molecular docking is a widely used approach. However, current scoring functions are poorly optimized for peptide ligands. Accordingly, PeptoGrid can fill the mentioned gap. The relevance of PeptoGrid ranking with a virtual screening of peptide libraries using angiotensin-converting enzyme and GABA_B_ receptor as targets has been investigated. A reasonable agreement between the computational and experimental data suggests that PeptoGrid is suitable for discovering functional leads. The PeptoGrid software is available at https://github.com/aozalevsky/peptogrid [34].

Finally, Aminpour and colleagues presented an interesting review regarding the application of molecular modeling techniques in drug discovery. The current status of high-performance computing applications in the general area of drug discovery has been reviewed. In particular, after the introduction related to the computational methodologies was applied at atomic and molecular scales, three specific examples of implementation of these tools have since been reported. The first example describes in-silico modeling of the adsorption of small molecules to organic and inorganic surfaces, which may be applied to drug delivery issues. The second example involves DNA translocation through nanopores with major significance to DNA sequencing efforts. The final example offers an overview of computer-aided drug design, with some illustrative examples of its usefulness [35].

In conclusion, as Guest Editor, I would like to thank all the authors and co-authors for their relevant contributions to this Special Issue, all the reviewers for their work in evaluating the submitted manuscripts, and the editorial staff of Molecules for their kind assistance. This special issue is accessible through the following link: https://www.mdpi.com/journal/molecules/special_issues/Computational_Approaches_for_Drug_Discovery. 
